# Application of Modified Skin Stretching for Soft Tissue Defect Reconstruction in the Ankle and Foot: A Retrospective Report

**DOI:** 10.1111/os.14265

**Published:** 2024-10-16

**Authors:** Xiaqing Yang, Yuping Liu, Weixing Wang, Xue Fang, Wang Zhang, Changhuan Liu, Xin Wang

**Affiliations:** ^1^ Department of Orthopaedic Trauma and Microsurgery Zhongnan Hospital of Wuhan University Wuhan China; ^2^ Hubei Clinical Medical Research Center of Trauma Microsurgery Wuhan China; ^3^ Department of Anesthesiology Sichuan University West China Second University Hospital Chengdu Sichuan China; ^4^ Department of Orthopaedic Badong People's Hospital Enshi China

**Keywords:** ankle and foot, skin and soft tissue defect, skin stretching

## Abstract

**Objective:**

The failure rate of foot and ankle soft tissue defect reconstruction with flap is relatively high, often posing a significant burden on patients. The aim of this study is to explore the effectiveness of repeated stretch sutures in repairing skin and soft tissue defects of the ankle and foot.

**Methods:**

Twenty‐three patients with ankle and foot skin and soft tissue defects were retrospectively analyzed between February 2016 and February 2019. Sutures were repeatedly stretched every 3–5 days. Local skin grafting was performed if necessary after wound surfaces disappeared or exposed tendons and bones were covered by soft tissue. Wound healing time, postoperative healing area, Vancouver Scar Assessment Scale, sensation, and function of the new skin were evaluated.

**Results:**

Healing time was 17–35 (24.43 ± 5.29) days. Ten patients wholly healed, and 13 healed by approximately 70.08% ± 6.59%. The Vancouver Scar Assessment Scale average score was 2.83 ± 1.19 points, of which 15 cases were excellent (0–3 points) and 8 cases were good (4–7 points). The sensation and function of the new skin after repair were equivalent to those of normal skin after the last follow‐up.

**Conclusions:**

Applying repeated tension sutures on the skin and soft defects of the ankle and foot reduced the skin graft area and decreased complex high‐risk surgical flaps' use and transplantation area.

## Graphical abstract

We used repeated tension sutures to repair skin and soft tissue defects in the ankle and foot to reduce the area of skin grafting, thus avoiding complex and high‐risk surgical flap operations.

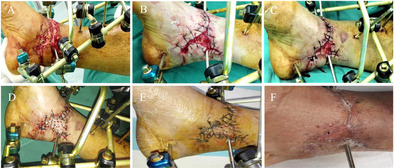

## 1 Introduction

The healing and functional repair of wounds and soft tissue caused by significant skin and soft tissue defects after trauma is a recognized problem in modern medicine. Common problems include pain, itching, visible reticular patterns, functional injuries caused by scar contraction, and hypertrophy [1, 2]. The foot and ankle are common clinical trauma sites, and injuries are often accompanied by a tendon or bone exposure, which can easily cause infection [3, 4]. Currently, these injuries are mainly treated by flap surgery. However, the soft tissue in the foot and ankle is scarce, leading to possible increased failure rate, delayed union, poor appearance, and more use of traditional flap transplantation. In addition, flap surgery on the foot and ankle is highly technical and sometimes requires a secondary surgery, which increases follow‐up treatment difficulty and postoperative pain [4, 5, 6, 7, 8]. Thus, it may result in chronic wounds, causing a significant burden on patients and the health care system [9, 10]. Therefore, clinical achievement of healing of significant skin and soft tissue defects has received great attention.

The skin can easily double its initial area while its thickness remains unchanged [11, 12, 13]. Skin stretching helps wound healing through two main mechanisms: mechanical creep and stress relaxation [14, 15, 16, 17]. When an external force is applied, the skin gradually stretches over time, and the tension gradually relaxes. When this is cyclical, the deformation of the skin will lead to a permanent stretch, and cyclical mechanical force may more effectively stimulate skin tissue growth [18, 19, 20, 21, 22]. New skin maintains the same thickness and preserves the same mechanical properties as natural tissue [15]. Mechanical stretching of the skin under certain conditions could promote the expansion of the wound skin and repair the skin soft tissue defect in situ [23, 24, 25, 26].

Various devices have previously been proposed to promote wound healing. K wires [27, 28], lacing apparatuses [29], Wisebands [30], and Hirshowitz devices [31] have been used clinically; however, most foot and ankle injury defects are irregular, and existing tools are only suitable for regular wounds and limited joint units. This equipment limitation has led to the technology above not being widely used for skin and soft tissue defects in the foot and ankle [32].

In recent years, we have reduced the use of skin stretching devices and have instead closed soft tissue defects around the foot and ankle adjacent to healthy skin by repeated pulling. This technique can be applied to any wound size or location without using a Kirschner wire or other accessory device. This simple operation could reduce the implementation of traditional flap surgery, narrow the skin graft area, and relieve the severity of the patient's pain and psychological pressure. It is highly feasible and has sound clinical effects.

Therefore, the purposes of this study were twofold: (i) to investigate the clinical outcomes of modified stretch sutures in repairing skin and soft tissue defects of the ankle and foot and (ii) to introduce the critical points and explore the advantages of this technique.

## 2 Patients and Methods

This retrospective study included 23 patients with skin and soft tissue defects of the ankle and foot who were admitted to our hospital between February 2016 and February 2019. The inclusion criteria were as follows: (i) patients with the presence of skin and soft tissue defects of the foot and ankle who underwent repeated stretch sutures in our hospital; and (ii) complete case data. The exclusion criteria were as follows: (i) follow‐up time less than 6 months; (ii) patients could not tolerate the treatment; and (iii) patients with severe microcirculation disease.

This study protocol conformed to the ethical guidelines of the 1975 Declaration of Helsinki and was approved by the Ethics Committee of Zhongnan Hospital of Wuhan University (2020060). Both the doctors and patients were aware of the treatment procedure.

### 2.1 Surgical Methods

Prior to thorough debridement, local or epidural anesthesia was administered to patients depending on the skin defect surface area, level of pollution, and patient's pain tolerance. If the patient suffered from a tibial fracture, it was set with an external fixation device. Patients with severe trauma, such as degloved skin or severe pollution, underwent vacuum sealing drainage before repeated stretch suturing to ensure adequate blood supply around the defect and sufficient healthy skin on both sides of the wound to maintain the stretching force; then, a stretch suture was performed.

Routine anti‐infective treatment was performed after distraction suture. The treatment of the wound before repeated distraction suture is also disinfected and cleaned according to the above method, and the operation methods and critical points of distraction suture are as follows: (i) The healthy skin with minor tension in the middle of the wound and good blood supply is selected as the needle entry point, and according to the shape of the wound, select the needlepoint on the opposite side corresponding to the skin adjacent to the wound edge. (ii) The margin of the suture needle is selected according to the number of the surrounding soft tissue and the location of the wound, generally 1–2 cm; the margin of the thinner part of the limb such as the toe is slightly smaller. (iii) The needle distance is evenly divided according to the edge tension of the wound, and it is determined according to the skin relaxation, blood supply, wound size, and different parts after pulling and knotting; the needle distance of the wound with a larger defect area can be more significant, and the needle distance can be gradually reduced after the area is reduced. (iv) Suture depth to the subcutaneous fascia, avoid suturing and deep vascular, nerve, and tendon, and take more subcutaneous tissue as much as possible. (v) Stretch sutures adopt No. 7 silk thread and simple intermittent sutures with tension size to tie the surgical knot. The first single knot can tighten the soft tissue without affecting the skin color, avoiding excessive tension and causing skin cutting. (vi) Evenly divide the pulling force, tie the knot while suturing, and judge the next needle's best needle entry and exit point, gradually uniformly close the defect wound. (vii) Pairs of more extensive wounds can be removed and re‐sutured after multiple stitches to reduce the defect as much as possible. (viii) When stitching again 3–6 days later, stagger the last suture, the first suture tighten the skin knot, and then remove the last loose suture. 0.05% iodophor gauze was applied to the suture surface after suture, then covered with sterile gauze and cotton pad. The dressing was changed daily, or the wound was covered with negative pressure closed drainage (vacuum sealed drainage, VSD) technique. After staging the stretch suture to complete the closure of the wound, the wholly closed skin was sutured with a simple break or a vertical break pad valgus with 1/4 silk thread. All patients received intensive care, good rest, sensitive antibiotics, and a nutritious diet with a daily dressing change or VSD as required during treatment.

### 2.2 Evaluation of Wound Size and Curative Effect

Clinical symptoms of the wound, such as pain and secretion levels; the healing condition, including whether the wound was healed, the healing time, the healing area, and the functional and sensory properties of the new skin, were evaluated.

The main observation index was the wound area before and after treatment. Measuring the wound area with a ruler is the most commonly used method in clinical practice and was also used in this study. The wound areas were determined as follows:

Mainly circular wounds: wound area = radius^2^ × 3 × 141(*π*).

Other wound shapes: mean wound area = length × [(minimum width of the wound + maximum width of the wound)/2].

The change in wound area was expressed as the percentage of wound area reduction. Each patient's average wound area reduction was calculated using a computer digital plane instrument (ImageJ, NIH, Bethesda, MD) to compare the before and after stretch suture photographs. The calculation method was as follows:

([Wound area before treatment − wound area after treatment]/wound area before treatment) × 100%.

Wound healing refers to skin sensory function and blood supply. Vancouver Scar Scale (VSS) was used for evaluation (including color, vascular distribution, softness, and thickness). The total score was 15 points, and the average score was 0, among which 0–3 was excellent, 4–7 was good, 8–11 was fair, and 12–15 was poor [33].

### 2.3 Statistical Analysis

The indicators are represented as mean ± SD and range and were calculated using IBM SPSS Statistics for Windows 23.0 (IBM, Armonk, NY).

## 3 Results

**FIGURE 1:**
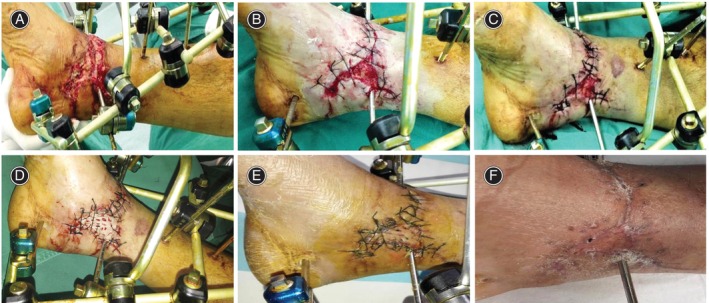
Male, 47 years old, the right foot was injured in a car accident. (A) Preoperative, the size of the wound is about 12.7 cm^2^, irregular shape, located at the medial ankle; (B) the first stretch suture after 3‐day debridement, the size of the wound was reduced by about 40%; (C) the second stretch suture performed 5 days after the first suture, the size of the wound was reduced by about 75%; (D) the wound was covered by skin graft 5 days after the second suture; (E) 7 days later after skin grafting, the skin graft is alive; and (F) follow‐up at 6 months after hospital discharge, the wound healed completely, and the skin graft area was almost the same color and texture as normal skin.

**FIGURE 2:**
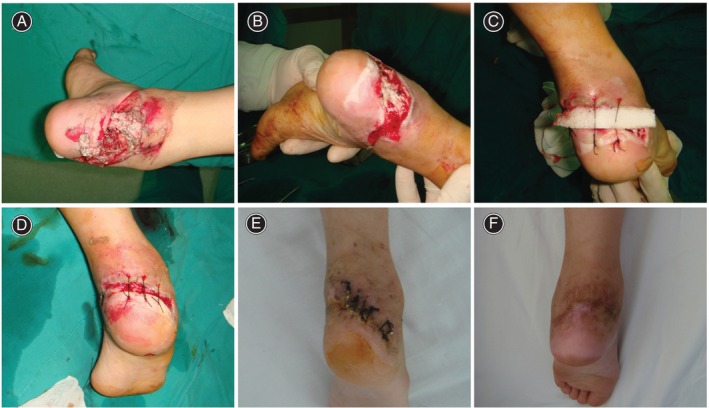
Female, 11 years old, twisted wound on the right ankle with tendon exposure. (A) Preoperative, the size of the wound is about 16.4 cm^2^, irregular shape; (B) after 3‐day vacuum sealing drainage, fresh granulation tissue can be seen on the wound with external leakage of Achilles tendon; (C) the first stretch suture after debridement, the size of the wound was reduced by about 40%; (D) the second stretch suture performed 5 days after the first suture, the size of the wound was reduced by about 70%; (E) the third stretch suture performed 5 days after the second suture, the wound was completely closed; and (F) follow‐up at 6 months after hospital discharge, the wound healed completely and the ankle joint functioned well.

The patients included in this study were aged 11–72 years, averaging 41.26 ± 16.64 years. The average ankle wound area was 10.11 ± 4.12 cm [2]. A tendon or bone exposure accompanied eight cases. Postoperative follow‐up was 6–8 months (7.04 ± 0.77 months). Operation time ranged from 0.3 to 0.9 h, with an average time of 0.51 ± 0.17 h. The wounds healed in 17–35 days, with an average healing time of 24.43 ± 5.29 days. All 23 patients' wounds completely healed—after repeated pulling and intermittent sutures in 10 patients and after repeated stretch sutures followed by an assisted skin graft in 13 patients. The average skin graft area was reduced by 70.08% ± 6.59% compared to the original defect area. There were three patients with wound infection, with an infection rate of 13.04%, including one case infected with *Staphylococcus aureus*, one with *Acinetobacter baumannii*, and one with *Enterobacter cloacae*, all of whom received antibiotic therapy and infection was controlled, and the progressive stretch suture was performed. At the last follow‐up, the patient's skin softness, sensory function, and blood supply after soft tissue defect healing were routine, and the patient was satisfied with the skin stretch effect. The average VSS score of 23 cases after healing was 2.83 ± 1.19 points, of which 15 cases were excellent and 8 cases were good (Figures 1 and 2).

## 4 Discussion

In this study, we improved the skin stretch device by repeating a point‐to‐point stretch suture. This significantly reduces the wound and skin graft areas in the ankle and foot and improves wound healing without using Kirschner wire or other accessory devices. All patients were satisfied with the results. This method could be applied to the treatment of defects in other parts of the body in the future.

### 4.1 The Advantages of Repeated Stretch Sutures in Repairing Skin and Soft Tissue Defects of the Ankle and Foot

The advantages of skin stretch sutures are as follows: (i) They allow for a simple operation without a particular skin stretch device that can be used for emergency treatment of open wounds with only basic surgical suture skills and high economic feasibility. (ii) Operation time is short, and local anesthesia can be used when tightening the sutures. It reduces patient risk while reducing the use of large‐area flap transplantation. Our technique has many advantages compared to the traditional large‐area flap transplantation with high technical content, long operation time, and more postoperative complications. (iii) The skin's color, texture, and sensory function grown by stretch sutures were the same as those of normal skin. (iv) We did not need to cut the normal skin in other areas, avoiding donor area and skin raft scarring. (v) The skin stretch device can provide healthy skin in an arthrosis depression, a load‐bearing area, or an irregular wound surface, all places where other devices are challenging to place, as well as can be used in other applications such as gout ulcer wounds of the ankle and diabetic foot ulcers. (vi) For severe or polluted trauma with missing skin, tendon, or bone exposure, it can effectively reduce the risk of infection from the exposed bone by promoting soft tissue cover and shortening wound closure time, thereby reducing the risk of amputation.

### 4.2 The Optimum Repeated Pulling Tension

The management of skin tension in surgical applications is a delicate process that requires adherence to existing standards while also making adjustments based on specific circumstances. Although some studies have provided reference standards for skin tightness, stretching frequency, and force, there is no one‐size‐fits‐all solution in practice. Each patient's skin characteristics are different, including elasticity, thickness, blood supply, and healing capacity, which makes standardization difficult [34]. Existing research has indicated that a force range of 0.5–4 kg is relatively safe during skin stretching. However, this is a general guideline and may not be suitable for all types of skin or surgical situations. For example, the skin of younger patients may be more elastic than that of older patients, while skin that has been affected by disease or scarring may require completely different handling [31]. Excessive skin stretching can increase the risk of a range of potential complications. Local vascular damage and skin thinning are two major issues that not only affect the healing of the surgical area but can also lead to wound dehiscence, infection, edge necrosis, and delayed healing. Therefore, the force and direction of stretching must be carefully designed to prevent damage to the blood supply and structural integrity of the skin.

To achieve the best results, the direction and force of stretching need to be adjusted according to the specific surgical requirements. Surgeons need to consider several factors, including:

- Natural skin tension lines (Langer's lines): Cutting and suturing along the natural texture lines can generally reduce tension and scarring.
- Skin elasticity: Understanding skin extensibility in different directions helps apply the appropriate force during stretching.
- Patient characteristics: Age, health status, and skin type all affect the skin's healing capacity and tolerance to tension.

With technological advancements, such as 3D imaging technology and realistic model simulations, surgical planning can more accurately meet individual needs. Additionally, the development of smart devices and sensors for real‐time monitoring of skin tension in surgical areas may open new avenues for improving surgical outcomes.

In summary, although there's currently no unified best practice for managing skin tension, by understanding the needs of different patients and surgical requirements, surgeons can more effectively tailor their techniques to optimize treatment results.

### 4.3 The Effect of Blood Supply to the Skin During Repeated Stretch Sutures

The effect of skin blood supply during traction requires additional attention. Pulling the skin produces repeated acute ischemia that can cause gradual recovery and degeneration of blood vessels [6, 35]. In this procedure, the blood supply of the normal skin was disturbed by the pulling; however, the physiological function of the skin was maintained without skin necrosis. There have been cases of wound necrosis and poor healing using this technique; however, they were unrelated to the nature of skin pulling and attributed to a decrease in the blood supply to the wound margin [32]. Therefore, when assessing whether a patient is an ideal candidate for skin pulling, the quantity and activity of the surrounding normal skin and soft tissue should be considered. If the patient's blood circulation [36] or overall health condition is poor, even within the safety threshold required to operate, they may not tolerate stretching. They could develop skin necrosis or other complications. We believe the skin tension suture suits patients with excellent skin blood supply and soft tissue elasticity without apparent inflammation or swelling. By repeatedly applying intermittent and controlled pull around the wound edge, healthy skin can be obtained without damaging the blood supply or the quality of the skin.

### 4.4 Limitation

There are also some limitations in this study. The number of cases in this study is small and it is a retrospective study. In the later stage, a large sample multi‐center prospective study is planned to further verify the effectiveness of this treatment method. And a disadvantage of this technique is that tension adjustments are made in practical applications based on observations of the wound, the patient's tolerance to stretching, and the surgeon's clinical experience; therefore, the approach's success is majorly dependent on the surgeon's skill and experience.

## 5 Conclusions

In this study, repeated tension sutures and auxiliary skin grafts were used to repair soft tissue defects in the ankle and foot. The appearance and sensory function at the wound sites were restored, the area of skin transplantation was significantly reduced, and early closure of critical structures such as nerves or tendons was observed. The procedure had a satisfactory clinical effect and was a proper auxiliary wound closure method worthy of popularization in clinical foot and ankle wound repair surgery. The skin's tolerance to different pull powers and compensation and improvement of the microcirculation around the wound should be evaluated in further studies.

## Author Contributions

**Xiaqing Yang:** conceptualization, methodology, writing – original draft. **Yuping Liu:** conceptualization, methodology, writing – original draft. **Weixing Wang:** statistical analysis, software. **Xue Fang:** follow‐ups. **Wang Zhang:** clinical data curation. **Changhuan Liu:** methodology, investigation. **Xin Wang:** funding acquisition, project administration, supervision, resources, writing – review and editing.

## Conflicts of Interest

The authors declare no conflicts of interest.
